# Potential Therapeutic Effects of Psilocybin: A Systematic Review

**DOI:** 10.7759/cureus.30214

**Published:** 2022-10-12

**Authors:** Dev B Goel, Sarju Zilate

**Affiliations:** 1 Medicine and Surgery, Jawaharlal Nehru Medical College, Datta Meghe Institute of Medical Sciences, Wardha, IND; 2 Pharmacology, Jawaharlal Nehru Medical College, Datta Meghe Institute of Medical Sciences, Wardha, IND

**Keywords:** anxiety, depression, addiction, cancer, psychedelic, psilocybin

## Abstract

Psilocybin is a plant alkaloid that is derived from precursors of tryptamine and is present in many different types of mushrooms. It has been utilized by indigenous peoples of Central and South America for centuries in a ceremonial setting to promote spiritual experiences. Indigenous societies have long employed psilocybin and other 5-HT 2A agonist classic psychedelics in their rites. They were a focus in psychiatry in the middle of the 20th century as both experimental medicines and tools for studying brain function. Due to the fact that traditional psychedelics were being used for purposes other than medical research and in connection with the burgeoning counterculture by the late 1960s and early 1970s, these scientific investigations fell out of favor. However, thanks to a number of encouraging studies that validated the earlier research, interest in traditional psychedelics has surged among scientists in the 21st century. In this review, we examine therapeutic studies on psilocybin, the traditional psychedelic that has received the lion's share of recent attention. According to three controlled studies, psilocybin may reduce symptoms of depression and anxiety in the context of cancer-related psychological discomfort for at least six months after a single acute treatment for mood and anxiety disorders. Three months after two acute doses, individuals in a small, open-label study with treatment-resistant depression reported fewer depressive and anxiety symptoms. Small, open-label pilot studies on addiction have demonstrated encouraging success rates for alcohol and cigarette addiction. The review also briefly discusses the synthesis, mechanism of action, effects, molecular pharmacology, adverse effects, and contraindications of psilocybin.

## Introduction and background

Conventional hallucinogenic substances, such as psilocybin, mescaline, and dimethyltryptamine, have been used by various native social orders for a very long time, ordinarily in consecrated settings [1]. Mescaline, the vitally psychoactive part of the peyote cactus, was first disengaged by Arthur Heffter in 1897. However, it was after 1943, with the amalgamation as well as the revelation of the psychoactivity of the lysergic corrosive diethylamide (LSD), that the synthetics started to gain mainstream attention. Strong starter results and contemporary exploration of conventional hallucinogenics have reignited interest in the therapy for both malignant growth and fixation. Despite the fact that psilocybin was the subject of the majority of clinical research recently, LSD was the exemplary hallucinogenic particle that got most of the attention in past examinations [2-3].

When consumed, hallucinogenics - a group of psychedelic medications - cause mind flights, which are mental bends and reality-changing impacts. Dreams, mindset swings, sensations of separation, and derealization are experienced occasionally as visualizations [2]. In view of their pharmacological profiles, hallucinogenics can be grouped into four major hallucinogenics: serotonin 2A (5-HT2A) receptor agonists, empathogens or entactogens (mixed serotonin and dopamine uptake inhibitors and inducers), dissociative narcotic psychedelics [N-methyl-D-aspartate (NMDA) miscreants], and unusual energizers, which have an extensive neurotransmitter structural impact [3-5]. 

**Figure 1:**
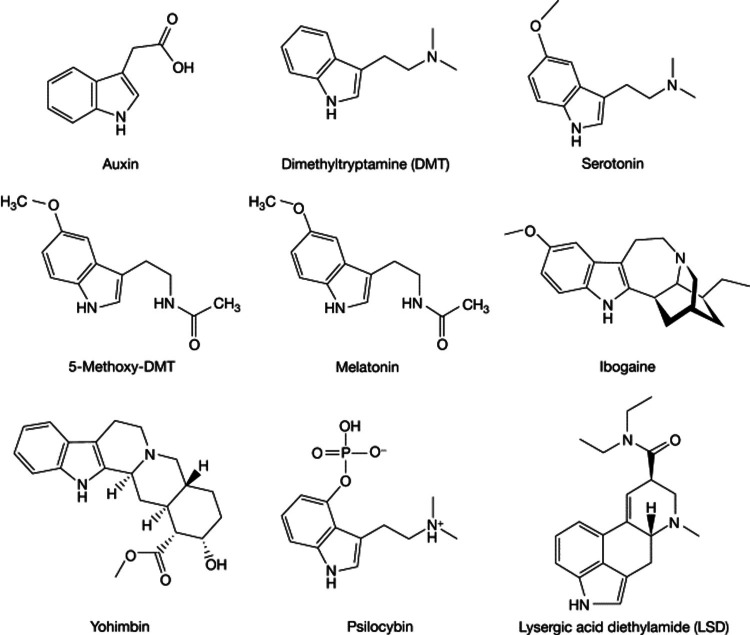
Structure of different hallucinogens* *[3] Auxin: artificially controls plant growth. DMT: N, N-Dimethyltryptamine (elicits intense visual alterations and hallucinations). Serotonin: key function in mood, sleep, and digestion. 5-methoxy-DMT: improvement in anxiety and depression. Melatonin: treats circadian rhythm sleep disorders. Ibogaine: treats drug addiction. Yohimbine: treats erectile dysfunction. Psilocybin: for alcohol use disorder, anxiety, migraines, and PTSD. LSD: lysergic corrosive diethylamide (treats psychosomatic diseases) The author has uploaded the figure on the internet for free use

Figure 1 shows the structure of various hallucinogens [3].

## Review

Psilocybin

Psilocybin has been found in more than 100 distinct kinds of mushrooms, large numbers of which belong to the family Psilocybe. Psilocybin is a moderately minuscule substance with a tryptamine-enlivened structure [4]. Psilocin, which is believed to be the dynamic fixing in the focal sensory system, is created when the prodrug psilocybin goes through in vivo de-phosphorylation. Psilocybin's impacts, similar to those of other conventional hallucinogenics, appear to be overwhelmingly interceded by combative action at the receptor of 5-hydroxytryptamine (HT)2A. However, the 5-HT2A movement does not appear to make sense of its belongings altogether. The physiological toxicity and misuse capability of psilocybin are modest [4,6-8].

Psilocybin synthesis

Various cycles can be utilized to make psilocybin. One potential transformation pathway for psilocybin from L-tryptophan is displayed in the image below [4]. Prior to arriving at the foundational course in rodents, psilocybin is completely changed into psilocin [6]. Contrary to prevalent thinking, psilocybin is not the essential pharmacologically dynamic part of enchantment mushrooms; rather, psilocin is. Psilocin gives wizardry mushrooms their psychotomimetic impacts. A forerunner substance to psilocin is psilocybin [4].

**Figure 2:**
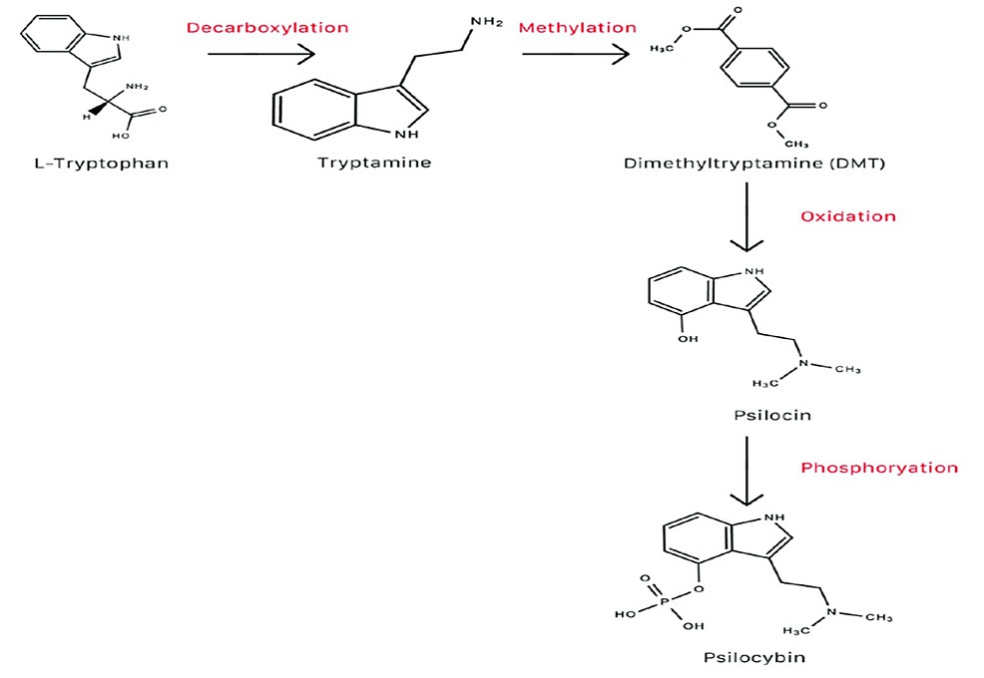
Transformation of L-tryptophan to psilocybin* *[6] The author has uploaded the figure on the internet for free use

Figure 2 shows the transformation of L-tryptophan to psilocybin [6].

Mechanism of action

Because of the enrollment of forward-looking hyper-frontality, psilocybin binds to 5-HT2A in the prefrontal cortex to exert its antidepressant and anxiolytic activities. The education or change of the typical medial prefrontal cortex's (mPFC) hyperactivity is one expected upper technique for action for psilocybin. The mPFC is in many cases overactive during depression [4]. Psilocybin's stimulant impacts are welcomed by adjusting the prefrontal and limbic cerebrum areas, along with the amygdala. In networks that cycle discernment and feelings, the amygdala is pivotal. A person with melancholy frequently turns out to be less delicate to close-to-home signs.

Furthermore, it has been observed that psilocybin binds to serotonergic receptor subtypes 5-HT2A and 5-HT1A with different affinities. Ketanserin, a 5-HT2A antagonist that lessens the effects of psilocybin, was utilized to demonstrate that psilocybin and psilocin cooperate with 5-HT2A receptors to incite psychotomimetic effects [6]. Cooperative organizations are separated and tactile capability networks are consolidated in psilocybin's difference in mind connections [9]. On a similar note, cooperation with input circles between the cortex and thalamus might be a conceivable method of activity for the psychotomimetic impacts of psilocybin [6,9].

Effects

Table 1 shows the psychological vs. somatic effects [10] of psilocybin.

**Table 1:** Psychological vs. somatic effects* *[10] The author has uploaded the figure on the internet for free use

Psychological effects in animals and humans [in medium dose (12–20 mg p.o.)]	Somatic adverse effects in humans (barely noticeable/secondary pharmacological effects)
Affective activation, hypnagogia, dreams, introspection, mystical-type experience, which predicts the success of the therapy and likelihood of benefits, hallucination, synaesthesia, alterations of time sense	At 8–12 mg p.o., i.m.; mydriasis, increased heartbeat frequency, decreased heartbeat frequency, hypotension, hypertension, nausea, increased reflex tendineae, decreased reflex tendineae, dysmetria, tremors. Similar results were noted in another trial at 0.11 mg/kg p.o. Other studies found no significant aberrations in the above-mentioned effects at 1.5 mg raised to 25 mg p.o. in three doses each day for 21 consecutive days

Molecular pharmacology

There are seven 5-HT receptors and their subtypes, out of which 5-HT2ARs are the most relevant for the functioning of psilocybin. The monoamine neurotransmitter 5-HT endogenously enacts 5-HT2ARs, which have a place with the wide group of heptahelical proteins called G protein-coupled receptors (GPCRs) [11]. The late Dolan Pritchett and others found 5-HT2ARs as basic film proteins in 1985, and when they were cloned in 1988, it was found that 5-HT2ARs are GPCRs. Comparative pharmacological attributes as far as ligand-restricting examples were found in examinations utilizing cloned 5-HT2ARs communicated in heterologous frameworks when contrasted with in situ-created receptors. High measures of 5-HT2AR courier ribonucleic corrosive (mRNA) were recognized in the mind using the Northern smudge examination, like prior examinations utilizing radioligand restricting techniques [12].

It is notable that 5-HT2ARs connect to the Gq group of heterotrimeric G proteins to actuate phospholipase C and phospholipase C breaks down cell membrane phosphatidyl inositol-4.5-biphosphate to yield inositol triphosphate and diacylglycerol. Subsequently, various signaling mechanisms are activated, including increased calcium influx and stimulation of protein kinase C (PKC), which thus sets off the actuation of PKC. Then, at that point, 5-HT2AR desensitization and various downstream effector pathways are incited by initiated PKC. All researched hallucinogenic substances to date are 5-HT2AR agonists, which open this route [11-13].

**Figure 3:**
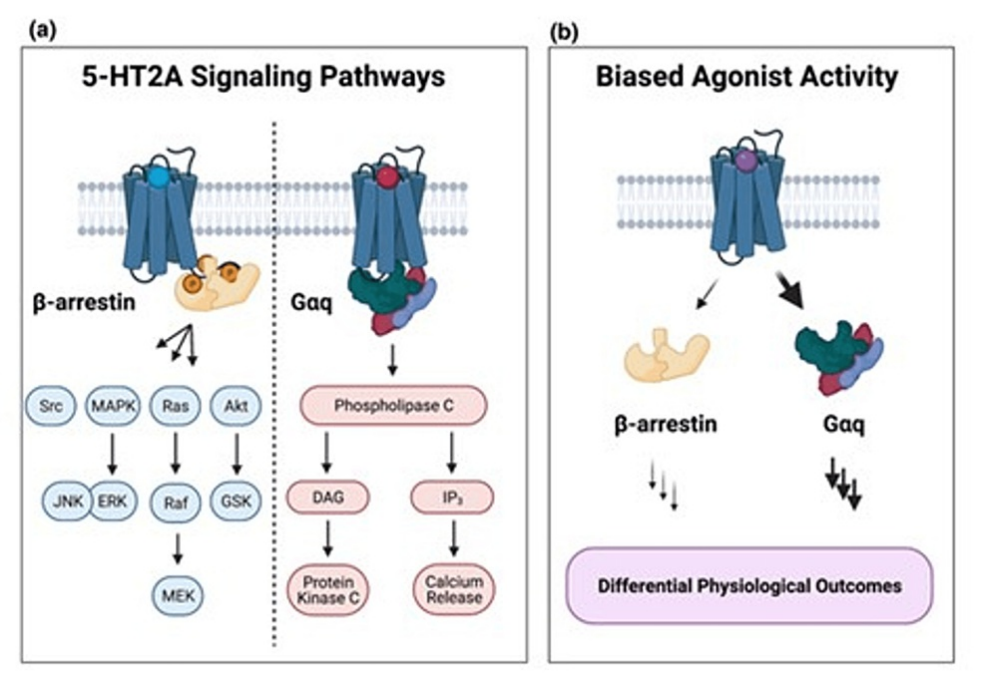
Relevance of 5-HT2AR signaling to psychedelic drug effects* *[13] (a) Diagram illustrating the intracellular signaling networks connected to the activation of the 5-hydroxytryptamine 2A receptor (5-HT2AR) and its effectors [11-13]. (b) Biased agonist activity of 5-HT2AR [11-13] The author has uploaded the figure on the internet for free use

Figure 3 shows the relevance of 5-HT2AR signaling to psychedelic drug effects [13].

Beginning examination on 5-HT2ARs exhibited enhancement in both rat and human cortex in light of radioligand restricting tests with non-particular 5-HT2AR antagonists radioligands (e.g., 3H-ketanserin, 3H-spiperone). Putative 5-HT2 receptors are amassed in synaptic films and intracellular compartments that resemble microsomal designs, as per subcellular fractionation studies. Various examinations utilizing receptor autoradiography have shown that cortical layer V in various species contains an improved populace of 5-HT2ARs. Various examinations utilizing receptor autoradiography have shown that cortical layer V in various species contains an upgraded populace of 5-HT2ARs [12-14].

Treatment model

In spite of most CNS medications, psilocybin was conveyed as a feature of organized psychotherapy for the remedial research facility examinations of the medication that will be examined later in this review [1]. This restorative setting incorporates screening to remove patients experiencing manic episodes, providing them with behavioral psychotherapy and subsequently utilizing them to advance enduring changes in conduct and disposition. Psilocybin, along with other psychedelics, is contraindicated in psychosis and patients experiencing manic episodes [2,15,16].

Cancer-Associated Psychological Distress

In a comparative study involving 12 patients with end-stage cancer, 0.2 mg/kg oral psilocybin showed a non-significant difference, compared to niacin [16,17].

In a clinical trial in patients with cancer, high-dose psilocybin improved depression as indicated by the Diagnostic and Statistical Manual of Mental Disorders, 4th edition (DSM-IV) criteria. Similarly, a six-month course of psilocybin reduced anxiety as demonstrated by the Hamilton Anxiety Rating Scale, the State-Trait Anxiety Inventory (STAI), the Hamilton Depression Rating Scale, and Beck Depression Inventory (BDI). While contrasting the two conditions earlier with the hybrid, this study found critical clinical enhancements in various regions, including diminished nervousness and trouble, and worked on the nature of life [16,17]. Customary hallucinogenics might be remedially successful for treating mental pain in the terminal and nonterminal malignant growth occurrences as well as in both disease and non-cancer ailment conditions [17].

Treatment-Resistant Depression

In a new small-scale, open-mark research, the effect of psilocybin was inspected beyond the setting of malignant growth. Psilocybin was administered orally to patients two times, once at 10 mg and once at 25 mg, separated by several weeks. When contrasted with pattern scores, burdensome side effects extensively diminished following multi-week and 90 days post-treatment as estimated by the Quick Inventory of Depressive Symptoms, the BDI, and different measures. For STAI-estimated uneasiness side effects, a similar outcome was observed [18-20].

Addiction

As early as the 1950s, studies were directed to see whether customary hallucinogenics could be utilized to treat addiction and substance dependence. A new meta-investigation of the six examinations - the only ones to haphazardly relegate members to LSD or another condition - found that LSD fundamentally diminished liquor abuse when contrasted with control bunches generally. Various observational examinations recorded reports of recuperation from dependence on liquor and different medications because of services including the utilization of exemplary hallucinogenics notwithstanding these early clinical investigations proposing possible adequacy of exemplary hallucinogenics in addiction treatment [21,22].

In an accessible research study, the authors of the current investigation gave psilocybin to 15 tobacco or nicotine-dependent smokers as a mental behavioral treatment for quitting smoking. After a year of follow-up, 10 out of 15 participants (67%) were subsequently found to be abstinent. A lengthy term that determined the middle value to be 2.5 years past the anticipated quit date resulted in the natural approval of nine out of the 12 individuals (75%) as abstinent. Psilocybin did not have any significant negative consequences [23]. An additional indication of the psychological mechanisms underlying psilocybin's effects on smoking cessation comes from a recent web-based study. This looked at 358 people who claimed to have stopped smoking or cut back after using a traditional hallucinogen. The subjects made it clear that there will be less severe emotional withdrawal side effects than in previous projects with conventional hallucinogens (like misery and cravings) [24,25].

A single-group proof-of-concept study to quantify the acute effects of psilocybin in alcohol-dependent participants and to provide preliminary outcome and safety data was conducted. Ten volunteers with DSM-IV-defined alcohol dependence received orally administered psilocybin in one or two supervised sessions in addition to motivational enhancement therapy and therapy sessions devoted to preparation for and debriefing from the psilocybin sessions. Participants' responses to psilocybin were qualitatively similar to those described in other populations. Abstinence did not increase significantly in the first four weeks of treatment (when participants had not yet received psilocybin) but increased significantly following psilocybin administration (p<0.05). Gains were largely maintained at the follow-up at 36 weeks. The intensity of effects in the first psilocybin session (at week four) strongly predicted change in drinking during weeks five to eight (r=0.76 to r=0.89) and also predicted decreases in craving and increases in abstinence self-efficacy during week five. There were no significant treatment-related adverse events. These preliminary findings provide a strong rationale for controlled trials with larger samples to investigate the efficacy and mechanisms of psilocybin [26,27].

Other Related Disorders

Obsessive-compulsive disorder (OCD): in a preliminary study, oral psilocybin's effects on people with urgent issues were researched in nine subjects. As per the Yale-Brown Obsessive Compulsive Scale, each member had a huge side effect decrease during somewhere around one meeting. One member showed long-haul gains at the half-year follow-up [25,27].

Cluster headaches: psilocybin-containing mushrooms, in addition to LSD, may be useful in treating cluster headaches or stopping their recurrence, according to one published case series of 53 self-administered individuals. This is intriguing because the illness has a limited number of effective licensed medications, and the pain it causes is frequently severe and incapacitating [25,28].

Adverse effects, risks, and contraindications

Psilocybin is currently not an approved medication since it is thought that it has a critical potential to be used. Psilocybin abuse can bring about what is known as a "terrible excursion" in uncontrolled circumstances, for example, during diversions. This is an upsetting or even awful physical and close-to-home experience that incorporates transient psychosis, insanity, suspicion, changed visual discernment, outrageous trouble, dread, absence of coordination, derealization, depersonalization, paresthesia, fits of anxiety, horrible flashbacks, and different side effects of schizophrenia [4,28].

Psilocybin is said to have the best safety profile of any hallucinogenic substance generally. Psilocybin has low physiological toxicity; it is a schedule I substance due to high abuse potentiality, low maltreatment/habit-forming responsibility, safe mental responses, and no connected tenacious destructive physiological or mental impacts during or after use, as per millennia of narrative proof and contemporary logical studies [4,24]. Albeit unprecedented, auto-mutilation and self-destructive contemplations have been described in people with mental or social diseases in the wake of taking wizardry mushrooms. Another risk is the potential for deteriorating maniacal symptoms [4,24]. With the utilization of a medicinally controlled climate, an instructor pre-directing to empower the right quiet point of view, and adequate expert mental and physiological help, the adverse effects connected with psilocybin might be deflected or reduced [4,25].

Summary

The condition of late examination suggests that psilocybin has a high remedial potential. Three randomized, fake treatment-controlled preliminaries involving psilocybin for the therapy of malignant growth-related mental trouble address the latest progression in this field of review. The utilization of psilocybin to help break out of habits is still in its beginning phases of exploration, yet two open-name pilot studies, one treating liquor reliance and the other cigarette smoking, have shown great promise [29]. The latest psilocybin research, especially regarding the proof of enduring remedial impacts that might result from a solitary prescription organization, recommends that conventional hallucinogenic treatment might address a critical new field of medication that might one day help to free individuals from experiencing an assortment of potential disorders [28,30].

## Conclusions

Treatments using hallucinogenics may offer new vistas on pressing issues with present CNS medications. As exhibited by many years of various clinical exploration and millennia of narrative stories, psilocybin-assisted treatment might be feasible, powerful, toxicologically safe, physiologically enduring, and may have implications in the field of CNS medication. Before it can turn into a staple in the treatment of mental disorders, there are a few deterrents that should be overcome. The profoundly sensationalized worldwide history, the absence of normalized hallucinogenic/psilocybin-related demonstrative and remedial practices, especially in promoting "otherworldly encounters," which are pivotal to the outcome of psilocybin treatment, and the absence of bigger, more adequate double-blinded, randomized, clinical examinations to assess security, pharmacology, and portion reaction connections for every mindset and nervousness problem represent some of several limitations.

Future psilocybin-put-together neuropharmaceuticals may likewise focus on non-psychoactive analogs of psilocybin as well as non-psychoactive innovative variants of psilocybin drugs, individualized neuropharmaceuticals to address the extraordinary necessities of a given patient, mixed treatment utilizing psilocybin or psilocin and different medications like marijuana, conventional psychotherapy, and blend treatment. Concentrating on how psilocybin interfaces with other psyche-changing and non-mind-adjusting prescriptions to treat temperament and nervousness issues could likewise be fascinating.

## References

[1] Nichols DE, Johnson MW, Nichols CD (2017). Psychedelics as medicines: an emerging new paradigm. Clin Pharmacol Ther.

[2] Reiff CM, Richman EE, Nemeroff CB (2020). Psychedelics and psychedelic-assisted psychotherapy. Am J Psychiatry.

[3] Nichols DE (2016). Psychedelics. Pharmacol Rev.

[4] Carhart-Harris RL, Goodwin GM (2017). The therapeutic potential of psychedelic drugs: past, present, and future. Neuropsychopharmacology.

[5] Goldberg SB, Pace BT, Nicholas CR, Raison CL, Hutson PR (2020). The experimental effects of psilocybin on symptoms of anxiety and depression: a meta-analysis. Psychiatry Res.

[6] Lowe H, Toyang N, Steele B (2021). The therapeutic potential of psilocybin. Molecules.

[7] Li NX, Hu YR, Chen WN, Zhang B (2022). Dose effect of psilocybin on primary and secondary depression: a preliminary systematic review and meta-analysis. J Affect Disord.

[8] Kuypers KP (2020). The therapeutic potential of microdosing psychedelics in depression. Ther Adv Psychopharmacol.

[9] Johnson MW, Griffiths RR (2017). Potential therapeutic effects of psilocybin. Neurotherapeutics.

[10] Mithoefer MC, Grob CS, Brewerton TD (2016). Novel psychopharmacological therapies for psychiatric disorders: psilocybin and MDMA. Lancet Psychiatry.

[11] Slocum ST, DiBerto JF, Roth BL (2022). Molecular insights into psychedelic drug action. J Neurochem.

[12] Ling S, Ceban F, Lui LM (2022). Molecular mechanisms of psilocybin and implications for the treatment of depression. CNS Drugs.

[13] López-Giménez JF, González-Maeso J (2018). Hallucinogens and serotonin 5-HT2A receptor-mediated signaling pathways. Curr Top Behav Neurosci.

[14] Heuschkel K, Kuypers KP (2020). Depression, mindfulness, and psilocybin: possible complementary effects of mindfulness meditation and psilocybin in the treatment of depression. A review. Front Psychiatry.

[15] McClure-Begley TD, Roth BL (2022). The promises and perils of psychedelic pharmacology for psychiatry. Nat Rev Drug Discov.

[16] Dinis-Oliveira RJ (2017). Metabolism of psilocybin and psilocin: clinical and forensic toxicological relevance. Drug Metab Rev.

[17] Ross S (2018). Therapeutic use of classic psychedelics to treat cancer-related psychiatric distress. Int Rev Psychiatry.

[18] Kverno KS, Mangano E (2021). Treatment-resistant depression: approaches to treatment. J Psychosoc Nurs Ment Health Serv.

[19] Patra S (2016). Return of the psychedelics: psilocybin for treatment resistant depression. Asian J Psychiatr.

[20] Kvam TM, Stewart LH, Andreassen OA (2018). Psychedelic drugs in the treatment of anxiety, depression and addiction. Tidsskr Nor Laegeforen.

[21] Vargas AS, Luís Â, Barroso M, Gallardo E, Pereira L (2020). Psilocybin as a new approach to treat depression and anxiety in the context of life-threatening diseases-a systematic review and meta-analysis of clinical trials. Biomedicines.

[22] Bogenschutz MP, Johnson MW (2016). Classic hallucinogens in the treatment of addictions. Prog Neuropsychopharmacol Biol Psychiatry.

[23] de Veen BT, Schellekens AF, Verheij MM, Homberg JR (2017). Psilocybin for treating substance use disorders?. Expert Rev Neurother.

[24] Bates ML, Trujillo KA (2021). Use and abuse of dissociative and psychedelic drugs in adolescence. Pharmacol Biochem Behav.

[25] Mertens LJ, Preller KH (2021). Classical psychedelics as therapeutics in psychiatry - current clinical evidence and potential therapeutic mechanisms in substance use and mood disorders. Pharmacopsychiatry.

[26] Gill H, Gill B, Chen-Li D (2020). The emerging role of psilocybin and MDMA in the treatment of mental illness. Expert Rev Neurother.

[27] Vogel M, Strasser H, Thorens G (2020). Addictions (Article in French). Rev Med Suisse.

[28] Courault P, Demarquay G, Zimmer L, Lancelot S (2021). Cluster headache: state of the art of pharmacological treatments and therapeutic perspectives. Fundam Clin Pharmacol.

[29] Tylš F, Páleníček T, Horáček J (2014). Psilocybin--summary of knowledge and new perspectives. Eur Neuropsychopharmacol.

[30] De Gregorio D, Aguilar-Valles A, Preller KH, Heifets BD, Hibicke M, Mitchell J, Gobbi G (2021). Hallucinogens in mental health: preclinical and clinical studies on LSD, psilocybin, MDMA, and ketamine. J Neurosci.

